# A MIXED-METHOD SOCIAL NETWORK ANALYSIS OF LOW-INCOME DIVERSE OLDER VOLUNTEERS

**DOI:** 10.1093/geroni/igac059.1324

**Published:** 2022-12-20

**Authors:** Katy (Qiuchang) Cao, Holly Dabelko-Schoeny, Keith Warren, Mo-Yee Lee

**Affiliations:** The Pepper Institute on Aging and Public Policy and The Claude Pepper Center, Florida State University, Tallahassee, Florida, United States; Ohio State University, Columbus, Ohio, United States; The Ohio State University, Columbus, Ohio, United States; The Ohio State University, Columbus, Ohio, United States

## Abstract

Although the health benefits of volunteering among older adults are well established in gerontology, older migrants’ abilities and interests in social participation are hardly recognized. To address the gap, we collected focus groups and survey information in Russian, Khmer, Somali, Nepali, and English to understand the volunteering experiences, social networks, and feelings of loneliness among low-income diverse volunteers in the Senior Companions Program (SCP) in Columbus, Ohio (N=41). The grounded theory approach informed the qualitative analysis. Exponential Random Graph Modeling (ERGM) was utilized to identify statistically significant structural features in the volunteers’ network. Five major themes emerged from the focus groups: (1) Expanding and strengthening social networks through volunteering; (2) Experiencing and coping with loneliness; (3) Experiencing and managing the social impact of COVID; (4) Exploring and loving the program; (5) Social connections outside of the program. Graphs and preliminary ERGM results demonstrate that participants tend to form homophily-based relationships with other volunteers of the same gender (β=2.45, p< 0.001) and from the same country (β=4.86, p< 0.001). However, participants tend to form friendships with people from different racial (β= -1.12, p< 0.001) and different educational backgrounds (β = -0.88, p< 0.001). The tendency to reciprocate (β= 0.96, p< 0.001) and to form triads (β= 9.90, p< 0.001) are both positively significant in the networks. Findings imply that practitioners should attend to within- and cross-cultural relationships in programs for diverse older adults. Addressing language barriers and other sources of homophily may facilitate cross-cultural friendships.

